# Leprosy in England

**Published:** 1889-07-20

**Authors:** 


### Within the Wards.

### LEPROSY IN ENGLAND.

### Is it Contagious?

The leprosy scare, which, though foolish, was not unnatural, is now passing away, and an opportunity offers for communicating to the public much valuable and trustworthy information on the disease as it manifests itself among our fellow countrymen who have acquired it under various circumstances. Mr. Jonathan Hutchinson, F.R.S., one of the greatest living authorities on all affections of the skin, is publishing a series of papers in the *British Medical Journal*, the chief points of which cannot but be deeply interesting to all readers who value intelligent and accurate knowledge of the subject. In England inherited and indigenous leprosy are practically unknown. Almost every leper, even when the patient is English, has acquired the disease in a foreign and mostly in an Eastern country. English practitioners are placed at some disadvantage, inasmuch as the number of cases in this country is so small that a very large majority of medical men never have a single opportunity of seeing a leper during the whole of their professional career. In spite of this, the disease, because of its ancient and classical history, has been much studied, and very decided opinions are held about it. It is generally understood that the profession divides itself into two opposite camps, the "contagionists" and the "non-contagionists." Mr. Hutchinson, with that power of clear vision which is so characteristic of him, believes that there is much less difference of real opinion among medical men than even they themselves think. "The truth," he says, "is probably nearly this: No one denies the possibility of contagion . . . while all, or nearly all, doubt whether contagion takes any important share in the spread of the disease." That contagion can be artificially effected is practically beyond doubt, since a recent experiment by inoculation appears to have been entirely successful. But this does not prove, nor even make it probable, that accidental contagion is common or easy. Those who believe that a leprosy bacillus is the real cause of the disease are bound *ipso facto* to believe also that it may be contagious. But the question of practical importance is, under what circumstances is contagion really effected? "On this point," says Mr. Hutchinson, "the testimony of modern observers in leprosy districts is nearly unanimous. Neither in Norway, in Hindustan, or the West Indian Islands do nurses or surgeons fear to come in contact with lepers from day to day for years together. So far as contagion is concerned, hundreds of surgeons, well acquainted with the dreadful nature of the disease, daily encounter the same risks that Father Damien did. They do so, however, in the firm conviction that the risk is nothing." On the very high authority, then, of Mr. Hutchinson, it may be said that under ordinary circumstances, and when reasonable precautions are taken, leprosy is, for all practical purposes, a non-contagious disease.

### What Risks did Father Damien Run?

When Father Damien first went to Molokai it was generally believed that he went to certain death, and that from a loathsome and painful disease. Mr. Hutchinson believes this to be as far as possible from the truth. The risk of contagion from the lepers already there was, in fact, almost nothing. There was a risk, and particularly to a man not acquainted with the medical aspects of the subject and with approved methods of prophylaxis. Mr. Hutchinson emphasises the risks that arise from "food." If a man be unsuitably fed, and give himself up to careless habits of eating, dressing, bathing, sleeping, and so on; if, in fact, he be merely careless of his general health, then, in a leper district, the risk of leprosy for him becomes very great. What Father Damien's habits in respect of food and general self-care were we do not know. We do know, however, that many persons of deeply religious life are apt to be more than usually careless of bodily health. "When I visited the leper establishments in Norway some years ago," says Mr. Hutchinson, "I made diligent enquiry for instances of apparent contagion. I asked more especially whether strangers visiting Norway ever contracted it. Everywhere I was met with an emphatic negative; but everywhere I was told of one well-known exception. This exception was a certain German officer who had come to live in Norway and had become a leper; but, added my informants, 'he degraded himself, and lived among the fishermen as they did, and he deserved to get it.' The large leper establishments at Bergen and Molde were officered by healthy persons, and no one feared the disease." It must not be supposed that these statements in any way detract from the noble heroism of Father Damien's life, for it is practically certain that he held the popular ideas as to the risk of contagion when he first went to Molokai; and that he continued to hold those ideas to the end of his life. In what manner he actually acquired the disease it is impossible to say. But the probability is that the food which he was compelled to eat had much to do with it.

### Is Leprosy a Definite Disease?

What is leprosy? Is it a particular and well-defined disease, quite distinct and different from every other disease, or is it a condition of bodily weakness which shows itself in the skin in more or less similar ways? This is a fundamental question. According to Mr. Hutchinson, it is an absolutely distinct and definite disease, springing always from the same source, as corn from seed, and showing always the same clear, characteristic, and unmistakeable features. There are no varieties either in the disease or its cause. The different names given to it merely indicate different stages of the same thing. Whether it show itself in the Norwegian fisherman, the Hindu peasant, the Sandwich Islander, or the West Indian planter, it is always leprosy, and nothing else but leprosy. This fact of "sameness," without regard to climate, social position, or any other circumstance, Mr. Hutchinson regards as of extreme importance. It points, without fear of error, to a cause which is always and everywhere one and the same. The cause and the result being always the same, the disease must also be invariably the same. As Mr. Hutchinson says, "A person is either a leper or he is not; he either has the poison, and will of necessity experience its full and dire effects, or he is wholly free." Leprosy may be briefly defined as a definite disease which springs from a specific microbe, the bacillus of leprosy, which affects principally the true skin, which runs a definite course, and which sooner or later tends to a fatal termination.

### Where is the True Source of Danger?

The source of danger Mr. Hutchinson insists is to be looked for in the food which the people eat who ultimately become lepers. Climate, poverty, hardship, dirt, he considers, have nothing at all to do with it, except as ancillaries; as, indeed, it is difficult to conceive how they can have if the disease can only spring from the leprosy bacillus, which is as true and definite a seed as is the seed of the cabbage or the spore of the fern. Since, it is argued, the disease can begin without being inherited, and contagion can only be brought about with difficulty: since, moreover, it affects people in climates that are both hot and cold, and pays no respect to wealth or station, age or sex, but picks out one here and another there of those who live in certain districts and conform to certain local modes of life, it is a not unnatural conclusion that the disease must be the product of a very special kind of poison, that the poison is of rare occurrence, and that it must be received into the system in connection with food. This food hypothesis, Mr. Hutchinson contends, would cover all the facts, whilst any other that is conceivable would as promptly be confuted by one or more facts, the reality of which cannot be denied. The question, therefore, according to this highly-competent investigator, stands thus: The leprosy bacillus is in some sense the cause of the disease; the spores of that bacillus are probably present in certain kinds of food largely partaken of in certain districts. We have to find some particular articles of food common to all leprosy districts and used by all classes of the people, and wholly or partially disused in districts that are non-leprous. Certain facts which bear out the food hypothesis are given, one of which may be cited as a sample of the rest. In 1847 a Jewish woman was under care at the Hospital for Diseases of the Skin, suffering from tubercular leprosy in a most severe form. She was then forty-four years of age, and had just returned from Jamaica. She had been born in Portsmouth, and had lived in England until she was thirty-two years of age. Then she had gone to Jamaica and remained there eleven years. When she left England for Jamaica she was perfectly well; when she returned to England, eleven years later, she was a pronounced leper. After her return to England the leprosy continued for a time, and then finally disappeared. Mr. Hutchinson saw her in 1879. She was then 71 years of age, and in excellent health. It may be argued that the disease might have disappeared even if she had remained in Jamaica. But against this is to be set the fact, that though she had suffered with it for several years before leaving that island, no signs of improvement showed themselves until she had been resident for some years in England. During her stay in Jamaica her diet had consisted of beef, turtle, and fish in great plenty. She had also had an abundant variety of vegetables. The most important fact connected with this woman was that whilst she had eaten fish almost every day in Jamaica, she ate no fish at all after her return to this country. Twelve cases in all are cited by Mr. Hutchinson, and each of these cases he had seen and treated himself. It is remarkable that most of them had occurred in previously healthy persons, who had lived for a time in districts where leprosy is endemic, and that most of them had been in the habit of eating fish freely, and some of them had been particularly fond of prawns. From this considerable mass of facts, collected with great care during many years, it is abundantly evident that investigation should leave nothing undone which may help to establish or to confute the food hypothesis. At present such a line of investigation seems fuller of promise than any other which is before the medical profession or the world.

